# A Randomized Trial Comparing the Bowel Cleansing Efficacy of Sodium Picosulfate/Magnesium Citrate and Polyethylene Glycol/Bisacodyl (The Bowklean Study)

**DOI:** 10.1038/s41598-020-62120-w

**Published:** 2020-03-27

**Authors:** Shih-Ya Hung, Hong-Chang Chen, William Tzu-Liang Chen

**Affiliations:** 1 Graduate Institute of Acupuncture Science, China Medical University, Taichung, 40402 Taiwan; 2 Department of Colorectal Surgery, China Medical University Hospital, Taichung, 40447 Taiwan; 3 School of Medicine, College of Medicine, China Medical University, Taichung, 40402 Taiwan; 4 Department of Colorectal Surgery, China Medical University Hsinchu Hospital, Hsinchu, 30272 Taiwan

**Keywords:** Colonoscopy, Randomized controlled trials

## Abstract

Bowel cleansing is essential for a successful colonoscopy, but the ideal clearing agent and the volume have yet to be determined. A small-volume cleanser is important for patient compliance. This study aimed to compare the bowel cleansing efficacy, safety, tolerability, and acceptability of a 300-mL small-volume sodium picosulfate/magnesium citrate (PSMC) preparation-Bowklean with one 2-L polyethylene glycol (PEG)/bisacodyl-Klean-Prep/Dulcolax preparation under identical dietary recommendations. This multicenter, randomized, parallel-group, pre-specified noninferiority study enrolled 631 outpatients scheduled to undergo colonoscopy (Bowklean = 316 and Klean-Prep/Dulcolax = 315). After bowel preparation, an independent evaluator blinded to the subject’s treatment allocation rated the quality of the colon cleansing. Efficacy was evaluated using the Aronchick Scale and Ottawa Bowel Preparation Scale (OPBS). Safety was assessed by monitoring adverse events. Tolerability and acceptability were measured via a patient questionnaire. Bowklean was non-interior to Klean-Prep/Dulcolax in overall colon cleansing but was associated with significantly better preparation quality. Notably, Bowklean was associated with significantly greater tolerability and acceptability of bowel preparations than Klean-Prep/Dulcolax. Safety profiles did not differ significantly between the groups. Our data indicate that Bowklean is a more effective and better-tolerated bowel cleansing preparation before colonoscopy than Klean-Prep/Dulcolax. Bowklean may therefore increase positive attitudes toward colonoscopies and participation rates.

## Introduction

Colorectal cancer is the second leading cause of cancer death amongst men and women in the USA^1,2^. Colonoscopy is the most utilized and cost-effective method to screen a variety of diseases, such as colorectal cancer^3^. A bowel preparation must effectively remove all feces from the colon before colonoscopy and help to prevent potential complications from surgery^4,5^. During a colonoscopy, stool in the colon can prevent the surgeon from seeing the tissue that is being inspected and complicates attempts to introduce the scope into the rectum and colon^4,5^. Moreover, an empty bowel greatly reduces the risk of infection if the bowel is nicked during surgery^6^.

The ideal bowel-cleansing agent should be well tolerated by subjects, easily administered, inexpensive, and produce adequate clearance without allowing explosive gases to form^7^. The majority of bowel preparations are either polyethylene glycol (PEG)-based or hyperosmotic; many of these regimens are perceived as unpalatable or unpleasant by patients^1^. PEG-containing preparations (e.g., Klean-Prep, GoLYTELY) are large-volume (2–4 L), osmotically-balanced nonabsorbable solutions that act as purgatives to evacuate the intestine^8^. Hundreds of studies have been performed to compare the various methods of bowel preparation and a split-dose, large-volume PEG regimen is considered to be the current standard for effective cleansing^1^.

The high volume of PEG products (2–4 L) means that many people fail to complete their bowel preparation regimens, leading to suboptimal visualization of the colon. Rates of inadequate bowel preparation are generally reported as ranging between 10% and 20% when scales are used to assess colon cleanliness^9–11^. A split-dose PEG regimen significantly improved the percentage of patients with satisfactory colon cleanliness, significantly increased patient compliance, and significantly decreased nausea^12^.

A Canadian trial comparing four bowel cleansing regiments has shown that PSMC + M (300 mL magnesium citrate) had the highest tolerability when compared with 4 L PEG, 2 L PEG + bisacodyl (20 mg), and NaP (90 mL)^13^. In terms of cleansing efficacy, 2 L PEG + bisacodyl or PSMC + M were both as efficacious as 4 L PEG and superior to NaP for bowel preparation^13^. Bisacodyl dose >10 mg can cause abdominal cramping and ischemic colitis^14–16^. In 2011, the United States Food and Drug Administration withdrew the 2 L PEG bowel cleansing kit HalfLytely containing bisacodyl 10 mg tablets, due to safety concerns of ischaemic colitis and abdominal cramping compared with the same kit using only bisacodyl 5 mg^17,18^. In Taiwan, 2 L Klean-Prep/bisacodyl is most commonly used and PEG/ascorbic acid is not available. The introduction of a small-volume (300 mL) PSMC preparation Bowklean (Universal Integrated Corporation, Taiwan) prompted us to conduct a randomized, controlled, endoscopist-blinded study to compare the efficacy and safety of that preparation with a 2-L PEG solution Klean-Prep (Helsinn-Birex Pharmaceuticals Limited, Ireland) combined with bisacodyl 5 mg (Dulcolax, Boehringer Ingelheim, Germany).

Adequate bowel cleansing may be achieved through a variety of mechanisms and recommendations for diet and hydrations, which vary from center to center^8^. This study compared the small-volume PSMC product, Bowklean, with a split-dose, large-volume PEG product, Klean-Prep/Dulcolax, for cleansing efficacy, safety, acceptability, and tolerability under standardized dietary advice in a cohort of 631 Taiwanese patients undergoing outpatient colonoscopy.

## Materials and methods

### Study design and trial information

This randomized, active-controlled, evaluator- and endoscopist-blinded, multicenter phase III clinical trial (ClinicalTrials.gov Identifier: NCT01984008; first posted date: 14/11/2013) was conducted in China Medical University Hospital and Changhua Christian Hospital (Taiwan). The Institutional Review Board of China Medical University Hospital and Changhua Christian Hospital approved the study.

### Sample size calculation, subject information, and selection of study participants

**Figure 1:**
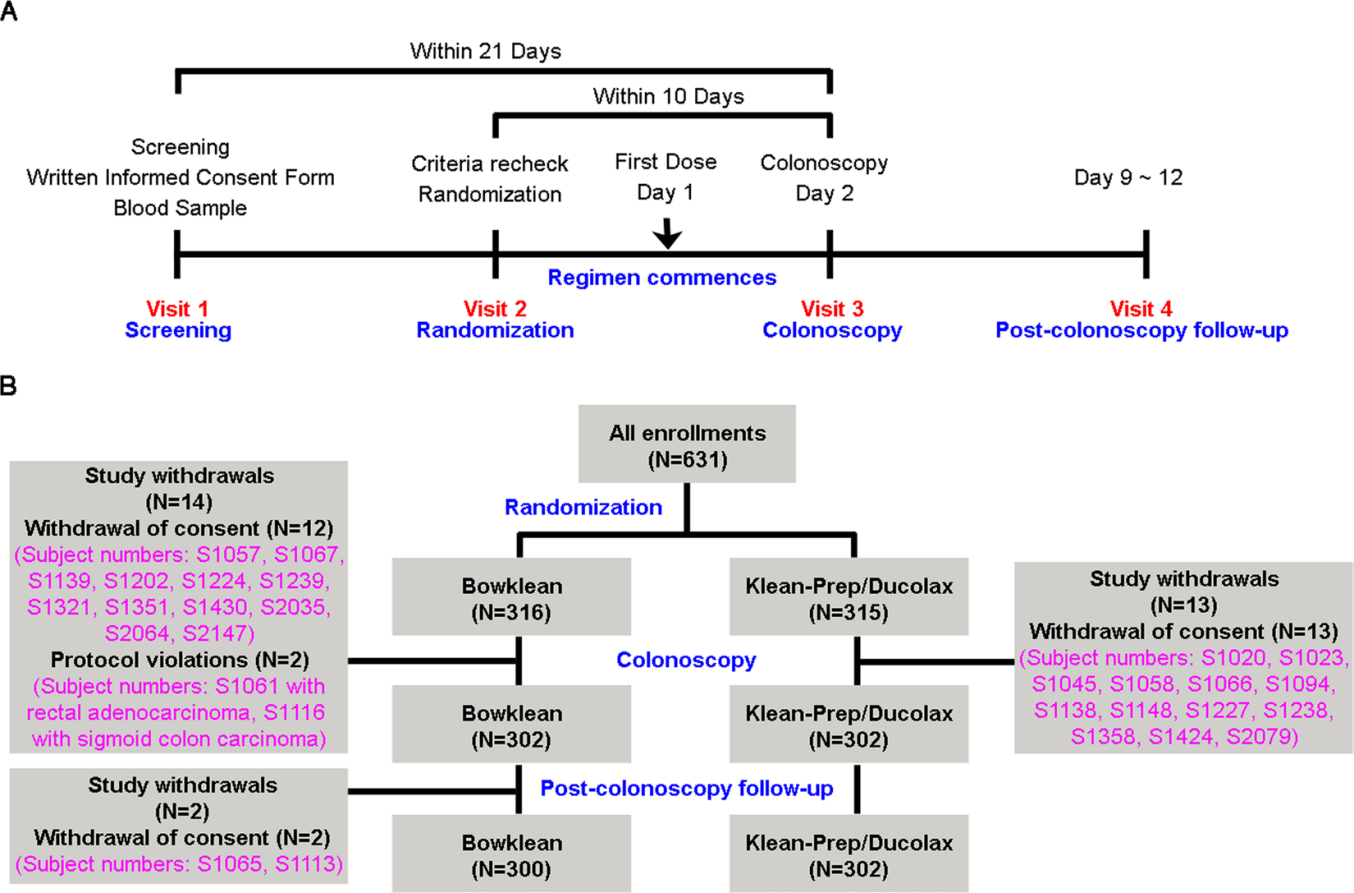
Flow diagram of our randomized trial comparing Bowklean with Klean-Prep/Dulcolax. (**A**) Flow chart of the study design and timetable. (**B**) Flow diagram of study subjects and subject numbers, with reasons for withdrawals.

The method used to calculate the sample size of this study is shown in Supplementary Table 1. A total of 631 outpatients were enrolled to obtain efficacy data. A flow chart detailing the study design and timetable of 4 visits (screening, randomization, colonoscopy, and post-colonoscopy follow-up) is depicted in Fig. 1A. The schedule of observations and procedures performed during each visit is detailed in Table 1. After undergoing screening for inclusion and exclusion criteria in Visit 1, patients completed informed written consent forms and underwent physical examinations that assessed vital signs, signs of pregnancy, liver and renal function, and serum electrolyte levels. The inclusion and exclusion criteria of this study are presented in Supplementary Table 1. All patients were enrolled between October 23, 2013 and March 24, 2014 and randomized to receive either the Bowklean (n = 316) or Klean-Prep/Dulcolax (n = 315) regimen. An overview of subject disposition (enrolment, randomization, study withdrawals, colonoscopy, and follow-up) is provided in Fig. 1B. Among randomized subjects, 604 patients underwent colonoscopy, 630 were in the full analysis set, 599 in the per-protocol set, and 630 in the safety analysis set. Supplementary Table 2 details patient disposition data for each treatment group for each analysis set, each visit, and study withdrawals. There were no significant between-group differences for baseline characteristics including sex, age, body mass index, weight, height, liver function, and vital signs (Supplementary Table 3).

**Table 1:** Schedule of the observations and procedures.

	Screening Visit	Randomization Visit*	Regimen Start	ColonoscopyVisit	Post-colonoscopy Follow-up Visit
Visit No.	1	2		3	4
Period (Day)	−19~ −1	−8~−1	1	2	9~12
Informed Consent	√				
Inclusion/Exclusion	√	√			
Medical History	√				
Vital signs	√	√		√	√
Pregnancy test (females only)	√				
Liver function	√^a^				
Renal function	√^a^			√^**b**^	√
Electrolytes	√^a^			√^**b**^	√
Randomization		√			
Dietary control			√		
Dosing day			√^**c**^	√	
Dietary card		√	√	√	
Subject questionnaire				√^**d**^	
Colonoscopy				√	
Aronchick Scale				√	
Ottawa Bowel Preparation Scale				√	
Bowel preparation compliance				√	
Concomitant medication		√	√	√	√
Solicited adverse events			√	√	
Unsolicited adverse events		√	√	√	√

*The screening and randomization visits could be conducted on the same day.

^a^After obtaining baseline laboratory data. The subject was randomized if s/he fulfilled the inclusion criteria. A total of 13 subjects were excluded from the study as they did not satisfy inclusion criteria.

^b^Laboratory tests were performed after the subjects completed ingestion of investigation products and before the colonoscopy procedure.

^c^The first dosing day was scheduled in the afternoon before the day of the colonoscopy.

^d^Tolerability and satisfaction of the preparation was determined by a standardized questionnaire administered on the day of the colonoscopy prior to the procedure.

### Low-residue dietary advice

During Visit 2, all subjects were rechecked for inclusion/exclusion criteria and vital signs and issued with a dietary card on the day before taking Bowklean or Klean-Prep/Ducolax (Table 1). The dietary card contained detailed instructions about dietary measures to be taken and the consumption of Bowklean or Klean-Prep/Dulcolax. An unblinded study coordinator recorded the following information: (a) standard dietary advice; (b) the start time, end time, and the number of bowel movements after the first regimen of study product before colonoscopy; (c) the number of cups of liquid consumed (solution and clear water). Subjects were instructed to give the completed dietary card and an empty bag of Bowklean or Klean-Prep/Dulcolax to the study coordinator on the day of the colonoscopy.

### Drug administration

Bowklean (two sachets; ingredients per sachet: 10.0 mg sodium picosulfate, 3.5 g magnesium oxide, 12.0 g anhydrous citric acid) was prepared immediately before each administration as follows: the contents of a single sachet of Bowklean were dissolved in 150 mL of water and stirred for 5 minutes. A split-dose regimen required the subjects to consume the first solution on the day before and the second one on the morning of the colonoscopy. The first sachet had to be taken during the evening before the day of the colonoscopy (about 6:00 PM), followed by 1,250 mL of clear liquids within 5 h. On the day of the colonoscopy, the second sachet of Bowklean was dissolved in 150 mL of water and consumed 5 h prior to the colonoscopy, then followed by 750 mL of clear liquids within a 2-h period.

The Klean-Prep/Dulcolax procedure (two sachets of Klean-Prep with 1 tablet of Dulcolax) required subjects to prepare Klean-Prep (ingredients per sachet: 59 g polyethylene glycol 3350, 5.685 g anhydrous sodium sulfate, 1.685 g sodium bicarbonate, 1.465 g sodium chloride, 0.7425 g potassium chloride, and 0.0494 g aspartame) immediately before each administration, by mixing one sachet with 1,000 mL of cold water, which had to be stirred thoroughly until the solution was clear. Subjects were instructed to consume one whole Dulcolax tablet (without chewing or crushing the tablet) containing bisacodyl 5 mg in the afternoon before the colonoscopy procedure. Starting at about 4 h after taking Dulcolax, subjects had to drink 2,000 mL of Klean-Prep solution over a 2-h period or approximately 250 mL every 15 min. Compliance with bowel cleansing was measured by subjects as the amount of study liquid consumed.

### Study design and treatment

The study treatment was blinded for both the colonoscopist and evaluator for the ary analysis. After screening (Visit 1), eligible subjects were randomly assigned to Bowklean or Klean-Prep/Dulcolax (Fig. 1B). Each subject’s participation was expected to last a maximum of 4 weeks (Fig. 1A). Study visits were at screening (Visit 1), randomization (Visit 2), colonoscopy (Visit 3), and at the 1-week post-colonoscopy follow-up (Visit 4) (Fig. 1A and Table 1). After bowel preparation, colonoscopy (Visit 3) was performed by an experienced colonoscopist. The quality of bowel cleansing seen during colonoscopy was rated and recorded in real-time after the colonoscopy by a completely blinded independent evaluator.

### Efficacy and safety outcome variables

Aronchick Scale and Ottawa Bowel Preparation Scale (OBPS) scores were used to grade colon cleanliness^19,20^. The primary endpoint of this study was the efficiency of colon cleansing as assessed by the Aronchick Scale, with success defined as “Excellent”, or “Good”^19^. “Inadequate” was defined as <90% of the mucosa observed, with the stool presenting as a solid or semisolid mixture that could not be suctioned or washed^19^. The OPBS was used to evaluate cleansing efficacy and patients’ acceptance and tolerance, which evaluated cleansing efficacy in the ascending, mid- (transverse and descending) and rectosigmoid segments of the colon^20^. OBPS score for each colon segment was graded by a 5-point scale as follows: Excellent, 0; Good, 1; Fair, 2; Poor, 3; and Inadequate, 4^20^. OBPS scores ranged from 0 to 14 (fluid scores, 0–2; and scores of ascending+mid+rectosigmoid segments, 0–12)^20^. The colonoscopist rated the overall fluid amount on a 3-point scale (where 0 = mild; 1 = moderate; 2 = large)^20^. This remnant fluid assessment score was added to the scores obtained for each colon segment to create a cumulative OBPS score. The secondary endpoint of the study was the patients’ safety, acceptance, and tolerance of the two bowel cleansing preparations. Safety was assessed by monitoring adverse events at each visit. All study procedures, including efficacy and safety measurements, were performed according to the schedule described in Table 1.

### Analysis of study populations

Efficacy was analyzed for the full and the per-protocol analysis sets, which were determined by an independent review of protocol violations and deviations before database lock. The full analysis set included all randomized subjects who had been assigned to either treatment and were used for supportive analyses of efficacy endpoints. The per-protocol analysis set included patients from the full analysis set who did not violate the study inclusion/exclusion criteria. The safety analysis set included all randomized subjects who had been assigned either study treatment. The patient disposition for each analysis set is presented in Supplementary Table 2.

### Statistical analysis

The statistical analysis in this study was implemented by a contract research organization, StatPlus Inc., which used SAS^®^ Version 9.4; the results were approved by Taiwan’s Food and Drug Administration. For the primary efficacy endpoint, the differences in success rates (excellent or good) were calculated using the Fisher’s exact test with associated exact 95% confidence intervals (CIs)^21^. Noninferiority was satisfied if the lower bound of the two-sided 95% CI for the difference in the success rate (Bowklean minus Klean-Prep/Dulcolax) was at least −9%. *P* values of less than 0.05 were regarded as statistically significant.

### Ethical approval

This study was reviewed and approved by the Internal Review Board (IRB) of China Medical University Hospital (CMUH102-REC2-066) and Changhua Christian Hospital (130809). Study methods were conducted in accordance with the IRB committee guidelines.

### Informed consent

Written informed consent was provided by all study participants.

## Results

### Study withdrawal rates

The study design and visit timetable (screening, randomization, colonoscopy, and post-colonoscopy follow-up) are depicted the flowchart in Fig. 1A. Study withdrawal rates were 5.1% for the Bowklean group and 4.1% for the Klean-Prep/Dulcolax group. The most commonly cited reasons for withdrawal were patient withdrawal of consent and protocol violation in the Bowklean group and patient withdrawal of consent in the Klean-Prep/Dulcolax group (Fig. 1B and Supplementary Table 2).

### Bowel cleansing efficacy

**Figure 2:**
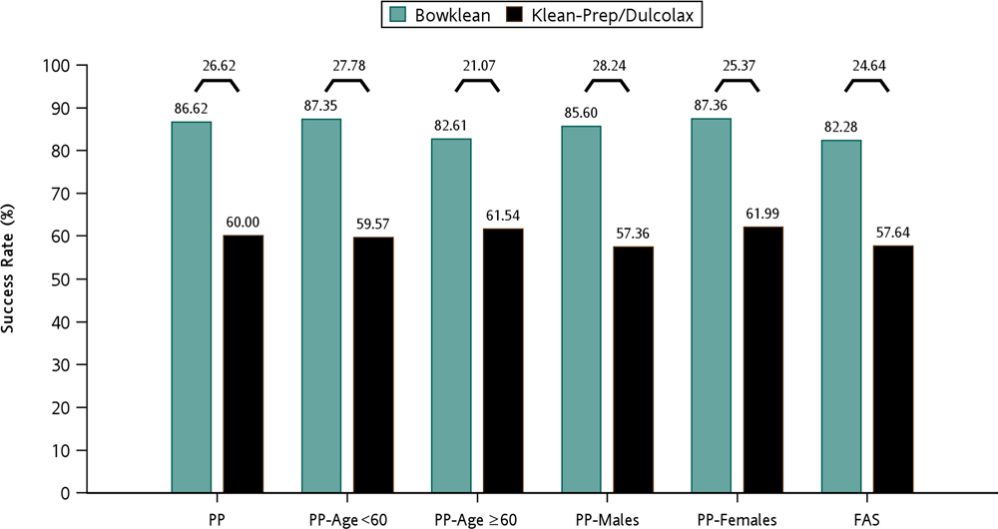
Summary of success rates as assessed by the Aronchick Scale for different patient populations of the per-protocol dataset. Results are shown by percentages and between-group differences (%) are shown at the top of each paired comparison. PP, the per-protocol analysis set; FAS, the full analysis set.

The primary variable of analysis was the bowel cleansing success rate, as assessed by the Aronchick Scale. In the per-protocol analysis set, the success rates (Excellent + Good) were 86.62% (n = 259) for Bowklean and 60.00% (n = 180) for Klean-Prep/Dulcolax (*P* < 0.0001; Table 2) which were consistent with the results in the full analysis set (Table 2). Table 3 presents the result of a robustness analysis of the dataset with logistic regression modeling including age and gender as covariates. The adjusted odds ratios were 4.296 in the per-protocol analysis set (95% CI, 2.861 to 6.452; *P* < 0.001) and 3.386 in the full analysis set (95% CI, 2.349 to 4.883; *P* < 0.001), indicating superior bowel cleansing quality with Bowklean compared with Klean-Prep/Dulcolax. In patients aged <60 years, success rates were 87.35% in Bowklean and 59.57% in Klean-Prep/Dulcolax (an adjusted between-group difference of 27.78%; *P* < 0.0001); corresponding values in those aged ≥60 years were 82.61% and 61.54%, respectively (an adjusted between-group difference of 21.07%; *P* = 0.0206; Fig. 2). In analyses by gender, success rates among males were 85.60% in the Bowklean group and 57.36% in the Klean-Prep/Dulcolax group (an adjusted between-group difference of 28.24%; *P* < 0.0001); corresponding values among females were 87.36% and 61.99%, respectively (an adjusted between-group difference of 25.37%; *P* < 0.0001; Fig. 2).

**Table 2:** Quality of cleansing using the Aronchick Scale.

Per-protocol analysis set			
Variables	Bowklean N = 299 (%)	Klean-Prep/Dulcolax N = 300 (%)	*P*-value
**Aronchick Scale**			**<0.0001**^**#**^
Excellent	157 (52.15%)	54 (18.00%)	
Good	102 (34.11%)	126 (42.00%)	
Fair	36 (12.04%)	109 (36.33%)	
Poor	2 (0.67%)	9 (3.00%)	
Inadequate	2 (0.67%)	2 (0.67%)	
**Success**			
Excellent+Good	259 (86.62%)	180 (60.00%)	
Exact 95% CI	82.23%–90.27%	54.21%–65.59%	
**Group Difference**	26.62%		**<0.0001**^**$**^
Exact 95% CI	18.88%–34.29%		
**Full analysis set**			
**Variables**	**Bowklean N** = **316**	**Klean-Prep/Dulcolax N** = **314**	***P*-value**
**Aronchick Scale**			**<0.0001**^#^
Excellent	158 (50.00%)	54 (17.20%)	
Good	102 (32.28%)	127 (40.45%)	
Fair	38 (12.03%)	110 (35.03%)	
Poor	2 (0.63%)	9 (2.87%)	
Inadequate	2 (0.63%)	2 (0.64%)	
No Assessment	14 (4.43%)	12 (3.82%)	
**Success**			
Excellent+Good	260 (82.28%)	181 (57.64%)	
Exact 95% CI	77.61%–86.33%	51.97%–63.17%	
**Group Difference**	24.64%		**<0.0001**^**$**^
Exact 95% CI	16.93%–32.05%		

^#^***P***-value was determined using the Mantel-Haenszel Test based on Ridit scores.

^$^***P***-value was determined using Fisher’s exact test.

**Table 3:** Quality of cleansing using the Aronchick Scale (robustness).

	Bowklean	Klean-Prep/Dulcolax	*P*-value
**Per-protocol analysis set**	**N** = **299 (%)**	**N** = **300 (%)**	
Excellent+Good	259 (86.62%)	180 (60.00%)	
Exact 95% CI	82.23–90.27%	54.21–65.59%	
Adjusted Odds Ratio*	4.296		**<0.0001**
Wald 95% CI	2.861~6.452		
**Full analysis set**	**N** = **316 (%)**	**N** = **314 (%)**	
Excellent+Good	260 (82.28%)	181 (57.64%)	
Exact 95% CI	77.61–86.33%	51.97–63.17%	
Adjusted Odds Ratio*	3.386		**<0.0001**
Exact 95% CI	2.349–4.883		

*Adjusted by age (continuous data) and gender (category data).

**Logistic analysis adjusted for age (continuous data) and gender (category data).

### Ottawa bowel preparation scale (OBPS) scores

**Figure 3:**
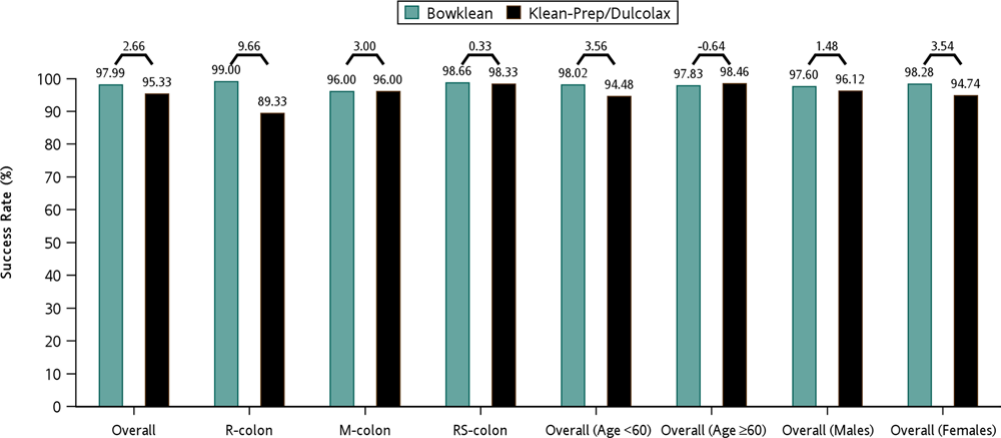
Summary of success rates as assessed by the Ottawa Bowel Preparation Scale (OBPS) for different patient populations of the per-protocol dataset. Success rates are presented by percentages (%) at the top of each bar and the between-group differences (%) are shown at the top of each paired comparison. R-colon, right colon; M-colon, mid-colon; RS-colon, rectosigmoid colon.

OBPS scores for the overall colon-cleansing efficacy in the per-protocol analysis set are presented in Table 4. Mean overall OBPS scores were 2.58 in the Bowklean group (n = 299) and 4.21 in the Klean-Prep/Dulcolax group (n = 300) (*P* < 0.0001; Table 4). Bowel cleanliness was rated as excellent in a significantly higher proportion of Bowklean recipients compared with Klean-Prep/Dulcolax recipients (41.14% vs 11.33%; *P* < 0.0001; Table 4). Success rates assessed by the OBPS did not differ significantly between Bowklean and Klean-Prep/Dulcolax preparations (97.99% vs 95.33%; between-group difference, 2.66%; 95% CI, –5.33% to 10.65%; Table 4). As the lower bound of 95% CI exceeded –9% in the per-protocol analysis set, the noninferiority of Bowklean was declared over Klean-Prep/Dulcolax. In age- and gender-adjusted analysis, Bowklean was associated with a higher success rate, with an odds ratio of 2.413 (Table 4), as well as a higher success rate for overall colon cleansing compared with Klean-Prep/Dulcolax (97.99% vs 95.33%; Fig. 3). The lower bound of the CI was >0% and the superiority of Bowklean was indicated in the cleansing of the right colon (Fig. 3). In subgroup analysis, noninferiority was demonstrated among patients aged <60 years and among females (Fig. 3).

**Table 4:** Quality of cleansing in the per-protocol analysis set using the Ottawa Bowel Preparation Scale (OBPS).

Variables	Bowklean N = 299 (%)	Klean-Prep/Dulcolax N = 300 (%)	*P*-value
**Mean Ottawa Scale**^**&**^			**<0.0001**^**@**^
Mean (SD)	2.58 (2.26%)	4.21 (2.26%)	
Median (Min, Max)	2.0 (0, 14)	4.0 (0, 14)	
**Ottawa Scale**			
Excellent	123 (41.14%)	34 (11.33%)	
Good	119 (39.80%)	138 (46.00%)	
Sufficient	51 (17.06%)	114 (38.00%)	
Poor	4 (1.34%)	12 (4.00%)	
Not Appropriate	2 (0.67%)	2 (0.67%)	
**Success**			
Excellent+Good+Sufficient	293 (97.99%)	286 (95.33%)	
Exact 95% CI	95.68–99.26%	92.29–97.43%	
Group Difference	2.66%		0.1093^**$**^
Exact 95% CI	−5.33–10.65%		
Adjust Odds Ratio*	2.413		0.0760*
Wald 95% CI	0.912–6.386		

^@^Two-sample *t*-test.

^#^Mantel-Haenszel Test based on score.

^$^Fisher’s exact test.

*Adjusted by age or gender.

^&^Incomplete colonoscopy be assigned as 14 (the worst case).

### Safety, acceptability, and tolerability results

Table 5 presents an overview of adverse events that occurred during the treatment period; 7 patients in each group reported at least 1 adverse event (*P* = 1.0000), nearly all of which occurred only once, and were transient and mild in severity. Serious adverse events were reported in 3 patients, all of whom received Klean-Prep/Dulcolax; 2 developed hemorrhoids and 1 experienced a colonic injury caused by colonoscopic perforation (Table 5). None of the adverse events were considered to be treatment-related. Table 6 shows the results of acceptability and tolerability in the per-protocol analysis set. Study participants reported that Bowklean was significantly easier to consume than Klean-Prep/Dulcolax (*P* < 0.0001) and to prepare according to the manufacturer’s instructions (*P* = 0.0151; Table 6). Significantly higher proportions of patients reported having an “Excellent” or “Good” experience with Bowklean (9.36% and 87.29%, respectively) compared with those administered Klean-Prep/Dulcolax (3.33% and 77.33%, respectively; *P* < 0.0001; Table 6). The taste of Bowklean was rated as “Excellent” or “Good” by significantly higher proportions of Bowklean recipients (27.09% and 68.23%, respectively) compared with Klean-Prep/Dulcolax recipients (1.00% and 40.00%, respectively (*P* < 0.0001 for both comparisons; Table 6). Of those assigned to Bowklean, nearly all (95.99%) reported that they would choose Bowklean again, compared with less than half (44.33%) of those randomized to Klean-Prep/Dulcolax (*P* < 0.0001; Table 6). Only 4.58% of the Bowklean group versus half of Klean-Prep/Dulcolax group (54.67%) claimed that they would refuse the preparation if offered it in the future (*P* < 0.0001; Table 6). Compliance rates were rated as excellent, good, medium, and poor in 92.1%, 3.2%, 0.3%, and 0.0% of Bowklean recipients, respectively; 4.4% were noncompliant (Supplementary Fig. 1). In the Klean-Prep/Dulcolax group, compliance rates were excellent, good, medium, and poor in 87.3%, 7.3%, 1.6%, and 0.0% of recipients, respectively; the noncompliance rate was 3.8% (Supplementary Fig. 1). The slightly higher rate of excellent compliance with Bowklean than with Klean-Prep/Dulcolax suggests a higher level of acceptance with Bowklean.

**Table 5:** Summary of all adverse events experienced by study participants (the safety analysis set).

CTACE	Bowklean N = 316 (%)	Klean-Prep/Dulcolax N = 314 (%)	*P*-value
Any adverse events	7 (2.22%)	7 (2.23%)	1.0000
Hematuria	1 (0.32%)	1 (0.32%)	1.0000
Hemorrhoids	0 (0.00%)	2 (0.64%)	0.2480
Abdominal pain	1 (0.32%)	0 (0.00%)	1.0000
Constipation	1 (0.32%)	0 (0.00%)	1.0000
Dyspnea	1 (0.32%)	0 (0.00%)	1.0000
Feeling cold	1 (0.32%)	0 (0.00%)	1.0000
Headache	1 (0.32%)	0 (0.00%)	1.0000
Large intestinal hemorrhage	1 (0.32%)	0 (0.00%)	1.0000
Menstruation irregularities	1 (0.32%)	0 (0.00%)	1.0000
Palpitations	1 (0.32%)	0 (0.00%)	1.0000
Periodontitis	1 (0.32%)	0 (0.00%)	1.0000
Seborrheic keratosis	1 (0.32%)	0 (0.00%)	1.0000
Swelling	1 (0.32%)	0 (0.00%)	1.0000
Calculus ureteric	0 (0.00%)	1 (0.32%)	0.4984
Colon injury	0 (0.00%)	1 (0.32%)	0.4984
Hydronephrosis	0 (0.00%)	1 (0.32%)	0.4984
Lower gastrointestinal hemorrhage	0 (0.00%)	1 (0.32%)	0.4984
Pyexia	0 (0.00%)	1 (0.32%)	0.4984
Spinal osteoarthritis	0 (0.00%)	1 (0.32%)	0.4984

*P*-values were determined by the Fisher’s exact test.

CTCAE, Common Terminology Criteria for Adverse Events.

**Table 6:** Acceptability and tolerability (the per-protocol analysis set).

Variables	Bowklean N = 299 (%)	Klean-Prep/Dulcolax N = 300 (%)	*P*-value
**Easy/difficult consumption**			**<0.0001**^**#**^
Very easy	55 (18.39%)	23 (7.67%)	
Easy	238 (79.6%)	258 (86.00%)	
Tolerable	5 (1.67%)	12 (4.00%)	
Difficult	1 (0.33%)	5 (1.67%)	
Very difficult	0 (0.00%)	2 (0.67%)	
**Able to consume per instruction**			0.0151^$^
Yes	299 (100.00%)	293 (97.67%)	
No	0 (0.00%)	7 (2.33%)	
**Overall experience**			**<0.0001**^**#**^
Excellent	28 (9.36%)	10 (3.33%)	
Good	261 (87.29%)	232 (77.33%)	
Fair	10 (3.34%)	48 (16.00%)	
Poor	0 (0.00%)	7 (2.33%)	
Bad	0 (0.00%)	3 (1.00%)	
**Taste**			**<0.0001**^**#**^
Excellent	81 (27.09%)	3 (1.00%)	
Good	204 (68.23%)	120 (40.00%)	
Fair	12 (4.01%)	86 (28.67%)	
Poor	2 (0.67%)	62 (20.67%)	
Bad	0 (0.00%)	29 (9.67%)	
**Request on subject’s own initiative**			**<0.0001**^**#**^
Yes	287 (95.99%)	133 (44.33%)	
No	12 (4.01%)	167 (55.67%)	
**Refuse the same preparation**			**<0.0001**^**#**^
Yes	14 (4.58%)	164 (54.67%)	
No	285 (95.32%)	136 (45.33%)	

^#^***P***-values were determined using the Mantel-Haenszel Test based on Ridit scores.

^$^***P***-values were determined using the Fisher’s exact test.

## Discussion

Colonoscopy is an important screening and therapeutic procedure for colon cancer^22^. The quality of bowel preparation impacts on colonoscopy success^22^. Bowel preparation is a complex undertaking, involving diet modifications and laxatives that are tailored to the individual patient^23^. A large number of bowel preparations are currently available, including PSMC, PEG, magnesium citrate, and NaP products^8^. In this study, using a low-residue diet as the control factor, we found that Bowklean demonstrated noninferiority over Klean-Prep/Dulcolax for overall colon cleansing and was associated with superior success rates, as determined by Aronchick Scale and OBPS scores. Bowklean also had a favorable safety profile, better tolerability, acceptability, and compliance. Compared with subjects assigned to Klean-Prep/Dulcolax, Bowklean recipients rated this preparation as easier to consume and were more willing to take the same preparation for any future colonoscopy.

Bowel preparations may cause adverse events. Between 1997 and 2002, the United States Food and Drug Administration received 100 reports of adverse events with PEG solutions, including 30 serious and 6 fatal events^24^. In the United Kingdom between 1995 and 2001, Ferring Pharmaceuticals Ltd. (United Kingdom) described 21 adverse events relating to the PSMC preparation (Picolax), including 5 serious adverse events, but no fatalities^25^. In the present study, treatment-related adverse events were reported less frequently by Bowklean recipients than by Klean-Prep/Dulcolax-treated patients. In both study groups, adverse events were mostly transient and mild in severity, similar to those reported with other commercially available bowel preparation reagents.

Ideally, colon cleansing preparations should have the following characteristics: (a) offer convenience for the patient; (b) be tolerable; (c) cause minimal distress; (d) be safe across different patient populations^26^. The American College of Gastroenterology recommends the use of a split-dose bowel regimen to improve the quality of colonoscopy and reduce the potential for suboptimal bowel preparation, which can lead to missed diagnoses, particularly of small lesions, and can increase costs due to aborted examinations or earlier rescreening because of poor visualization of the mucosa^9,10,27^. One report, consisting of 93,004 colonoscopies suggests that inadequate preparation quality hinders the detection of smaller lesions and has a negligible impact on the detection of larger lesions^10^. The small sample size in our study prevents any meaningful analysis of a relationship between bowel preparation quality and colonoscopic detection of suspected colonic neoplasia.

Of 25 studies that have compared PEG preparations with PSMC preparations, the PEG solutions differed (2–4 L), as did the methods of PSMC dosing (2 or 3 packages), and dosing strategies^21^. In the present study, the colon cleansing results with Bowklean are similar to those previously reported with PSMC (Prepopik)^28,29^. In 2 studies, each involving around 600 patients, split-dose administration of Prepopik achieved the primary endpoint (successful colon cleansing) and demonstrated noninferiority over single-dose 2 L PEG/bisacodyl^28,29^. Prepopik also demonstrated statistical superiority over the 2-L PEG preparation of-HalfLytely plus bisacodyl tablet bowel preparation kit (Braintree Laboratories, Inc, MA)^28,29^. In a study that included 341 patients, a PSMC preparation (Picoprep, Ferring B.V., The Netherlands) proved to be noninferior for efficacy and safety to an ascorbic acid-enriched PEG solution (Moviprep, Norgine, UK) plus bisacodyl^21^. In a clinical trial involving 68 patients, 3 sachets (16.5 g each) of PSMG preparation was found to be better tolerated, had significantly fewer side effects, and resulted in higher-quality bowel cleansing than a 3-L PEG preparation^30^. In 2012, a Korean study reported the outcomes of comparison between PSMC (Picolight, 94 Korean subjects) plus a low-residue diet with the standard bowel preparation of 4 L PEG solution (90 Korean subjects) on bowel preparation efficacy and patient satisfaction^31^. Bowel preparation with PSMC plus a low-residue diet enhanced colon cleansing and was better tolerated than 4 L PEG^31^. In the present study, all participants were Taiwanese and issued with standard dietary advice, as a means of reducing the effect of different dietary patterns.

The limitation of this study is that it was double-blinded, with the colonoscopist blinded as to individual treatment assignment (for performing colonoscopies) and an independent evaluator who was blinded for the primary analysis. Normally, two evaluators are required. This study employed only one evaluator to ensure that one fully trained evaluator in Aronchick Scale and OBPS scoring would evaluate each patient with the same criteria. To avoid bias, further study should employ two evaluators to compare the efficacy, tolerability, and safety of Bowklean with that of Klean-Prep/Dulcolax.

## Conclusion

Under standard low-residue dietary conditions, the PSMC preparation Bowklean demonstrated noninferiority over the PEG/bisacodyl preparation Klean-Prep/Dulcolax and achieved higher success rates in patients preparing for colonoscopy in Taiwan. Bowklean also had a favorable safety profile, was generally well tolerated and was more acceptable than Klean-Prep/Dulcolax. Bowklean may increase patients’ positive attitudes towards colonoscopy and motivate them to fully comply with all necessary bowel preparation procedures.

## Supplementary information

Supplementary information.

## References

[1] Rutherford CC, Calderwood AH (2018). Update on Bowel Preparation for Colonoscopy. Curr Treat Options Gastroenterol.

[2] Pan J, Xin L, Ma YF, Hu LH, Li ZS (2016). Colonoscopy Reduces Colorectal Cancer Incidence and Mortality in Patients With Non-Malignant Findings: A Meta-Analysis. Am J Gastroenterol.

[3] Sonnenberg A, Delco F, Inadomi JM (2000). Cost-effectiveness of colonoscopy in screening for colorectal cancer. Ann Intern Med.

[4] Rex DK (2006). Quality indicators for colonoscopy. Am J Gastroenterol.

[5] Committee ASOP (2015). Bowel preparation before colonoscopy. Gastrointest Endosc.

[6] Shah HA, Paszat LF, Saskin R, Stukel TA, Rabeneck L (2007). Factors associated with incomplete colonoscopy: a population-based study. Gastroenterology.

[7] Atkin, W. S. *et al*. Single blind, randomised trial of efficacy and acceptability of oral picolax versus self administered phosphate enema in bowel preparation for flexible sigmoidoscopy screening. *Bmj* 320, 1504–1508; discussion 1509 (2000). 10.1136/bmj.320.7248.1504 PMC27392 10834891

[8] Barkun A (2006). Commonly used preparations for colonoscopy: efficacy, tolerability, and safety–a Canadian Association of Gastroenterology position paper. Can J Gastroenterol.

[9] Froehlich F, Wietlisbach V, Gonvers JJ, Burnand B, Vader JP (2005). Impact of colonic cleansing on quality and diagnostic yield of colonoscopy: the European Panel of Appropriateness of Gastrointestinal Endoscopy European multicenter study. Gastrointest Endosc.

[10] Harewood GC, Sharma VK, de Garmo P (2003). Impact of colonoscopy preparation quality on detection of suspected colonic neoplasia. Gastrointest Endosc.

[11] Mitchell RM, McCallion K, Gardiner KR, Watson RG, Collins JS (2002). Successful colonoscopy; completion rates and reasons for incompletion. Ulster Med J.

[12] Kilgore TW (2011). Bowel preparation with split-dose polyethylene glycol before colonoscopy: a meta-analysis of randomized controlled trials. Gastrointest Endosc.

[13] Kao D (2011). A randomized controlled trial of four precolonoscopy bowel cleansing regimens. Can J Gastroenterol.

[14] Ajani, S., Hurt, R. T., Teeters, D. A. & Bellmore, L. R. Ischaemic colitis associated with oral contraceptive and bisacodyl use. *BMJ Case Rep* 2012, 10.1136/bcr-12-2011-5451 (2012). 10.1136/bcr-12-2011-5451 PMC4542499 22843752

[15] Baudet JS, Castro V, Redondo I (2010). Recurrent ischemic colitis induced by colonoscopy bowel lavage. Am J Gastroenterol.

[16] Lopez Morra HA, Fine SN, Dickstein G (2005). Colonic ischemia with laxative use in young adults. Am J Gastroenterol.

[17] US Food and Drug Administration, HHS. Determination That HalfLytely and Bisacodyl Tablets Bowel Prep Kit (Containing 4 Bisacodyl Delayed Release Tablets, 5 Milligrams) Was Withdrawn From Sale for Reasons of Safety or Effectiveness. National Archives. Federal Register.

[18] Clark RE (2013). Low-volume polyethylene glycol and bisacodyl for bowel preparation prior to colonoscopy: a meta-analysis. Ann Gastroenterol.

[19] Aronchick, C. A., Lipshutz, W. H., Wright, S. H., DuFrayne, F. & Bergman, G. Validation of an instrument to assess colon cleansing. *Am J Gastroenterol*. **94** (1999).

[20] Rostom A, Jolicoeur E (2004). Validation of a new scale for the assessment of bowel preparation quality. Gastrointest Endosc.

[21] Mathus-Vliegen EMH, van der Vliet K, Wignand-van der Storm IJ, Stadwijk JS (2018). Efficacy and Safety of Sodium Picosulfate/Magnesium Citrate for Bowel Preparation in a Physically Disabled Outpatient Population: A Randomized, Endoscopist-Blinded Comparison With Ascorbic Acid-Enriched Polyethylene Glycol Solution Plus Bisacodyl (The PICO-MOVI Study). Dis Colon Rectum.

[22] Bechtold ML, Mir F, Puli SR, Nguyen DL (2016). Optimizing bowel preparation for colonoscopy: a guide to enhance quality of visualization. Ann Gastroenterol.

[23] Hassan C (2013). Bowel preparation for colonoscopy: European Society of Gastrointestinal Endoscopy (ESGE) guideline. Endoscopy.

[24] Hookey LC, Depew WT, Vanner S (2002). The safety profile of oral sodium phosphate for colonic cleansing before colonoscopy in adults. Gastrointest Endosc.

[25] Periodic safety update report for Picolax. Report No. Report# FPL/Picolax/2/2001 (2001).

[26] Hsu CW, Imperiale TF (1998). Meta-analysis and cost comparison of polyethylene glycol lavage versus sodium phosphate for colonoscopy preparation. Gastrointest Endosc.

[27] Rex DK (2009). American College of Gastroenterology guidelines for colorectal cancer screening 2009 [corrected]. Am J Gastroenterol.

[28] Katz PO (2013). A dual-action, low-volume bowel cleanser administered the day before colonoscopy: results from the SEE CLEAR II study. Am J Gastroenterol.

[29] Rex DK (2013). Split-dose administration of a dual-action, low-volume bowel cleanser for colonoscopy: the SEE CLEAR I study. Gastrointest Endosc.

[30] Regev A (1998). Comparison of two bowel preparations for colonoscopy: sodium picosulphate with magnesium citrate versus sulphate-free polyethylene glycol lavage solution. Am J Gastroenterol.

[31] Kim YS (2014). Randomized clinical trial comparing reduced-volume oral picosulfate and a prepackaged low-residue diet with 4-liter PEG solution for bowel preparation. Dis Colon Rectum.

