# Supplementary material for: A Randomized Trial Comparing the Bowel Cleansing Efficacy of Sodium Picosulfate/Magnesium Citrate and Polyethylene Glycol/Bisacodyl (The Bowklean Study)
Source: Sci Rep. 2020 Mar 27;10:5604. doi: 10.1038/s41598-020-62120-w (PMC7101403; doi:10.1038/s41598-020-62120-w)
Supplement: Supplementary file 1 — Supplementary information. [file 41598_2020_62120_MOESM1_ESM.docx]

**Supplementary information**

***Title Pages***

**A Randomized Trial Comparing the Bowel Cleansing Efficacy of Sodium Picosulfate/Magnesium Citrate and Polyethylene Glycol/Bisacodyl (The Bowklean Study)**

Shih-Ya Hung^1, 2^, Hong-Chang Chen^2^, William Tzu-Liang Chen^2, 3, 4, *^

^1^Graduate Institute of Acupuncture Science, China Medical University, Taichung 40402, Taiwan

^2^Division of Colorectal Surgery, Department of Surgery, China Medical University Hospital, Taichung 40447, Taiwan

^3^College of Medicine, China Medical University, Taichung 40402, Taiwan

^4^Division of Colorectal Surgery, Department of Surgery, China Medical University Hsinchu Hospital, Hsinchu 30272, Taiwan

***Corresponding author**: William Tzu-Liang Chen, M.D., Division of Colorectal Surgery, Department of Surgery, China Medical University Hospital, No.2, Yude Road, Taichung 40447, Taiwan. Tel: +886-4-22052121 ext. 1639; Fax: +886-4-22070569. E-mail address: wtchen@mail.cmuh.org.tw

**Short running head:** Bowklean for bowel preparation

**Keywords:** Bowel preparation, colonoscopy, clinical trial, polyethylene glycol, sodium picosulfate/magnesium citrate

**Supplementary Table 1. The sample size calculation, inclusion and exclusion criteria of the study.**

| **Sample size calculation** |
| --- |
| The sample size calculation was determined by assuming a 9.0% non-inferiority margin, at least 80% power, and a one-sided significance level of 0.025. Based on these assumptions, a total of 280 subjects per group were required to verify the non-inferiority of the Bowklean group to the Klean-Prep/Dulcolax group. To allow for departure in these assumptions, it was anticipated that up to 5% of randomized subjects were ineligible for the clinical per protocol population. Therefore, approximately 600 subjects were planned to be recruited to provide 560 per protocol evaluable subjects. |
| **Inclusion criteria** |
| (a) age between 20 and 80 years; (b) men or non-pregnant women scheduled to undergo an elective colonoscopy; (c) subjects were able and willing to complete the entire procedure and to comply with study instructions; (d) written informed consent was obtained prior to study participation. |
| **Exclusion criteria** |
| (a) subjects with acute surgical abdominal conditions (e.g., acute obstruction and perforation); (b) active (acute/exacerbation of/severe/uncontrolled) inflammatory bowel disease; (c) colon disease (history of colonic cancer, toxic megacolon, toxic colitis, idiopathic pseudo-obstruction, or hypomotility syndrome); (d) gastrointestinal disorder (active ulcer, outlet obstruction, gastric retention, gastroparesis, or ileus); (e) any prior colorectal surgery in the past 3 months, excluding appendectomy, hemorrhoid surgery or prior endoscopic procedures; (f) history of upper gastrointestinal surgery (gastric resection, gastric banding, or gastric bypass); (g) severe chronic constipation; (h) ascites; (i) renal insufficiency (serum creatinine >1.5 times the upper limit of normal creatinine clearance <30 mL/min); (j) uncontrolled angina and/or myocardial infarction within the previous 3 months before randomization; (k) congestive heart failure or uncontrolled hypertension; (l) participation in an investigational study within 60 days prior to receiving study medication; (m) any clinically significant laboratory value at screening, including pre-existing electrolyte abnormality, that was deemed by the investigator as potentially affecting the study evaluation; (n) hypersensitivity to any ingredient in the study medication. |

**Supplementary Table 2. Summary of subject disposition**

|  | **Bowklean**  **N=316** | **Klean-Prep/Dulcolax**  **N=315** | ***P*-value** |
| --- | --- | --- | --- |
| **Randomized** | 316 (100.0%) | 315 (100.0%) | － |
| **Safety analysis set** | 316 (100.0%) | 314 (99.7%) | 0.4992 |
| Subject excluded from the safety analysis set | 0 | 1 |  |
| Exclusion reason |  |  |  |
| Others* | 0 | 1 |  |
| **Full analysis set** | 316 (100.0%) | 314 (99.7%) | 0.4992 |
| Subject excluded from the full analysis set | 0 | 1 |  |
| Exclusion reason |  |  |  |
| Others* | 0 | 1 |  |
| **Per-protocol analysis set** | 299 (94.6%) | 300 (95.2%) | 0.8640 |
| Subject excluded from the per-protocol analysis set | 17 | 15 |  |
| Primary exclusion reason |  |  |  |
| Violated study exclusion criteria | 4 | 0 |  |
| No colonoscopy performed | 12 | 12 |  |
| Received prohibited medication during the treatment period | 1 | 1 |  |
| Others* | 0 | 2 |  |
| **Visit 1 (Screening)** | 316 (100.0%) | 315 (100.0%) | － |
| **Visit 2 (Randomization)** | 316 (100.0%) | 315 (100.0%) | － |
| **Visit 3 (Colonoscopy)** | 302 (95.6%) | 302 (95.9%) | 1.0000 |
| **Visit 4 (Follow-Up)** | 300 (94.9%) | 302 (95.8%) | 0.7045 |
| **Withdrawal from study**  **Primary reason for withdrawal** | 16 (5.1%) | 13 (4.1%) | 0.7045 |
| Withdrawal of consent | 16 (4.4%) | 13 (4.1%) | － |
| Violation and/or significant deviation of study protocol | 2 (0.6%) | 0 (0.0%) | － |

*P*-values were determined using the Fisher’s exact test.

*One subject (S1023) did not take the assigned medication and subsequently re-screened as S1091.

**Supplementary Table 3. Summary of demographic and baseline characteristics (the full analysis set)**

| Item/Category | Bowklean  N=316 (%) | Klean-Prep/Dulcolax  N=314 (%) | *P*-value |
| --- | --- | --- | --- |
| Sex |  |  | 0.8083 |
| Male | 129 (40.8%) | 132 (42.0%) |  |
| Female | 187 (59.2%) | 182 (58.0%) |  |
| Age (years) |  |  | 0.0851 |
| Mean (SD) | 47.7 (12.0) | 49.4 (12.8) |  |
| Median | 48 | 50.0 |  |
| (Min, Max) | (20,76) | (21,80) |  |
| Age group |  |  | 0.1005 |
| Less than 60 years | 266 (84.2%) | 248 (79.0%) |  |
| 60 years or older | 50 (15.8%) | 66 (21.0%) |  |
| Body Mass Index (kg/m^2^) |  |  | 0.5928 |
| Mean (SD) | 24.1 (3.9) | 24.3 (3.6) |  |
| Median | 23.7 | 23.9 |  |
| (Min, Max) | (16.4, 44.5) | (16.8, 38.4) |  |
| Weight |  |  | 0.9931 |
| Mean (SD) | 63.95 (12.83) | 63.96 (12.28) |  |
| Median | 62.0 | 62.4 |  |
| (Min, Max) | (40.0, 111.0) | (42.9, 115.0) |  |
| Height |  |  | 0.4012 |
| Mean (SD) | 162.52 (8.97) | 161.95 (8.27) |  |
| Median | 161.0 | 161.4 |  |
| (Min, Max) | (141.8, 186.0) | (143.0, 184.0) |  |
| Alanine aminotransferase |  |  | 0.9263 |
| Mean (SD) | 24.42 (15.38) | 24.31 (13.19) |  |
| Median | 21 | 20.05 |  |
| Aspartate aminotransferase |  |  | 0.8529 |
| Mean (SD) | 25.37 (10.34) | 25.24 (8.16) |  |
| Median | 23 | 23 |  |
| (Min, Max) | (12, 110) | (13, 77) |  |
| Pulse |  |  | 0.5164 |
| Mean (SD) | 73.19 (11.48) | 72.62 (10.36) |  |
| Median  (Min, Max) | 70  (55,113) | 72  (52, 108) |  |
| Systolic blood pressure |  |  | 0.9465 |
| Mean (SD) | 124.44 (15.53) | 124.52 (13.72) |  |
| Median | 122 | 122 |  |
| (Min, Max) | (96,168) | (97, 164) |  |
| Diastolic blood pressure |  |  | 0.7134 |
| Mean (SD) | 71.66 (9.86) | 71.37 (9.87) |  |
| Median | 70 | 70 |  |
| (Min, Max) | (52, 102) | (52, 100) |  |
| Body temperature |  |  | 0.1774 |
| Mean (SD) | 36.15 (0.22) | 36.13 (0.19) |  |
| Median | 36.2 | 36.1 |  |
| (Min, Max) | (35.6, 37.0) | (35.6, 37.0) |  |

*P*-values were determined using the Fisher’s exact test.

**Supplementary Figure 1**

**
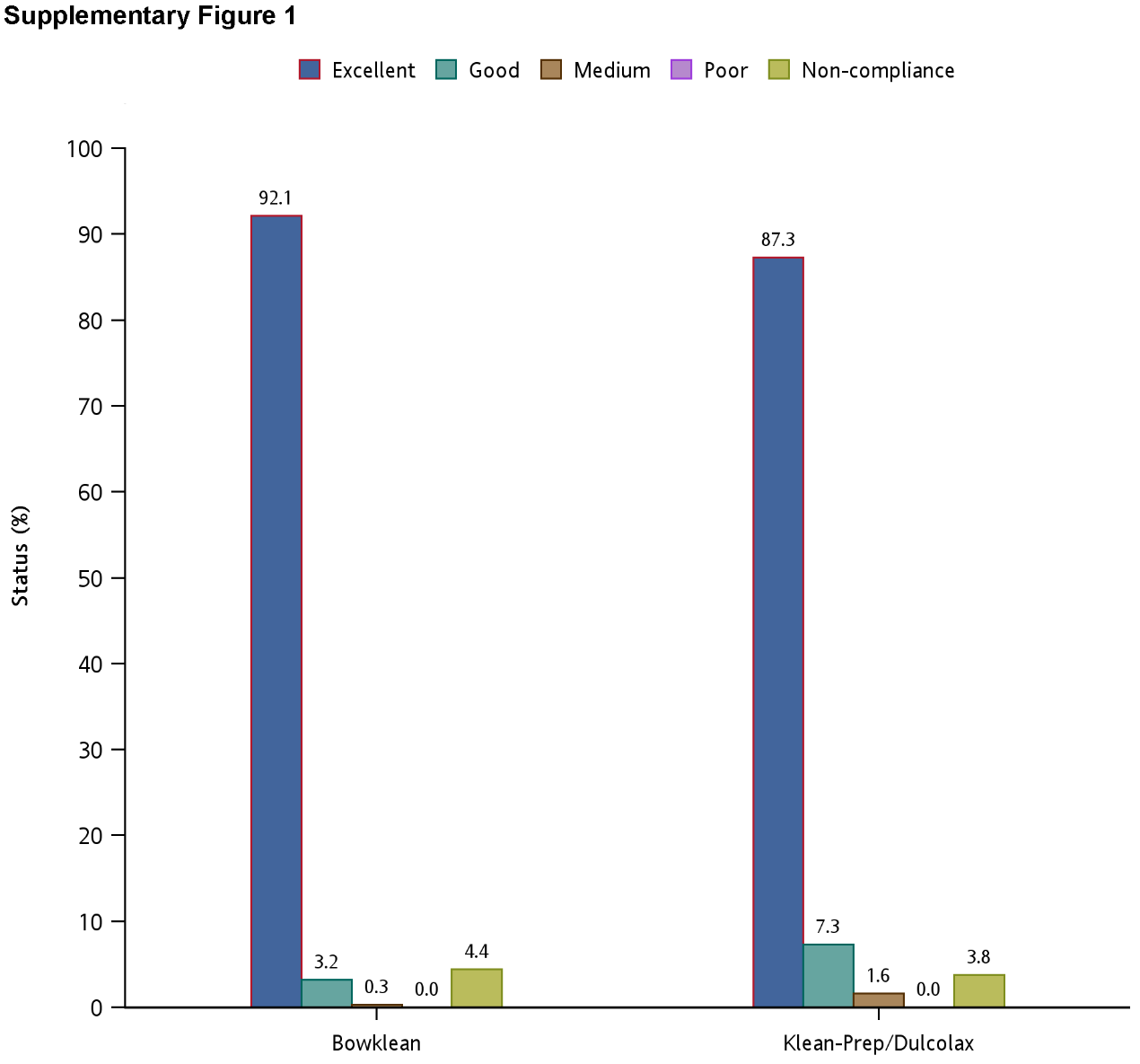
 Supplementary Figure 1.** Summary of treatment compliance. Safety analysis set was used for data analysis; results are presented as percentages.
